# Longitudinal analysis of complete blood count parameters in advanced‐stage lung cancer patients

**DOI:** 10.1111/1759-7714.13642

**Published:** 2020-09-17

**Authors:** Livia Rojko, Zsolt Megyesfalvi, Eszter Czibula, Lilla Reiniger, Vanda Teglasi, Zsolt Szegedi, Zoltan Szallasi, Balazs Dome, Judit Moldvay

**Affiliations:** ^1^ 1st Department of Pulmonology National Koranyi Institute of Pulmonology Budapest Hungary; ^2^ Department of Tumor Biology National Koranyi Institute of Pulmonology Budapest Hungary; ^3^ Department of Thoracic Surgery Semmelweis University and National Institute of Oncology Budapest Hungary; ^4^ Division of Thoracic Surgery, Department of Surgery Comprehensive Cancer Center, Medical University of Vienna Vienna Austria; ^5^ 4th Department Pulmonology National Koranyi Institute of Pulmonology Budapest Hungary; ^6^ 1st Department of Pathology and Experimental Cancer Research Semmelweis University Budapest Hungary; ^7^ Hungarian Brain Research Program, 2nd Department of Pathology Semmelweis University Budapest Hungary; ^8^ Computational Health Informatics Program Boston Children's Hospital, USA, Harvard Medical School Boston Massachusetts USA; ^9^ Danish Cancer Society Research Center Copenhagen Denmark

**Keywords:** Advanced‐stage lung cancer, complete blood count, organ metastases

## Abstract

**Background:**

Metastatic lung cancer is a debilitating disease, but with the advances in immunotherapy, therapeutic options have vastly increased. Numerous complete blood count parameters (CBC) have been described as easily accessible biomarkers that might predict response to immunotherapy. However, to date, no comprehensive study has been performed on the longitudinal changes of these parameters during cancer progression.

**Methods:**

The clinicopathological variables and CBC parameters of 986 advanced stage lung cancer patients were retrospectively analyzed. Blood tests were performed as part of the routine checkup and the results were recorded at the time of the diagnosis of the primary tumor, the diagnosis of brain or bone metastases, and also during the last available follow‐up.

**Results:**

In the experimental subcohort, 352 and 466 patients were diagnosed with brain and bone metastases, respectively. The control group consisted of 168 patients without clinically detectable or other distant organ metastases. In our longitudinal analyses, we found significantly decreasing absolute lymphocyte count (ALC: *P* < 0.001), and significantly increasing absolute neutrophil count (ANC: *P* < 0.001) levels in all patient subgroups, irrespective of histopathological type and metastatic site. Interestingly, patients with brain metastases had significantly descending‐ascending platelet count (PLT) trendlines (*P* < 0.001), while the bone metastatic subgroup exhibited significantly ascending‐descending trendlines (*P* = 0.043).

**Conclusions:**

Significantly decreasing ALC, significantly increasing ANC and fluctuating PLT levels may be found in brain and bone metastatic lung cancer patients during disease progression. Our findings might contribute to improve personalized healthcare in this devastating malignancy.

**Key points:**

**Significant findings of the study:**

- Significantly decreasing ALC, and significantly increasing ANC levels can be found in advanced‐stage lung cancer patients during disease progression
- Patients with brain metastases have descending‐ascending PLT trendlines, while patients with bone metastases exhibit ascending‐descending trendlines during disease progression

**What this study adds:**

- The descending values for ALC, and the ascending mean values for PLT and ANC, might be suggestive of poor response to second‐ or third‐line immunotherapy in advanced‐stage lung cancer patients.
- The current study might help to improve patient selection and treatment strategies for brain and/or bone metastatic lung cancer patients.

## Graphical abstract

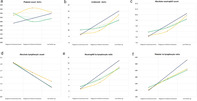

In our longitudinal analyses, we found significantly decreasing ALC, and significantly increasing ANC levels in advanced‐stage lung cancer patients during disease progression. Interestingly, patients with brain metastases have descending‐ascending PLT trendlines, while patients with bone metastases exhibit ascending‐descending trendlines during disease progression. The current study might help to improve patient selection and treatment strategies for brain and bone metastatic lung cancer patients.

## Introduction

Metastatic lung cancer, the most frequently diagnosed malignancy and leading cause of death from cancer worldwide, is a debilitating disease that results in a high burden of symptoms and poor quality of life.^1^, ^2^ The preferential sites for metastasis in lung carcinomas irrespective of their histopathological type are the brain, bones, adrenal glands and contralateral lung.^3^, ^4^, ^5^ About 25%–40% and 30%–40% of patients with lung cancer develop brain and bone metastases, respectively.^6^ Although the prognosis for these extensive‐stage patients with known brain or bone metastases is still poor with a median survival of less than one year, no comprehensive treatment strategies have as yet been developed.^6^, ^7^

In the past decades, adequate treatments have been limited for patients with distant organ metastases whose disease progressed after first‐ or second‐line chemotherapy, but in recent years, several immunotherapeutic agents have been developed which have proven to be beneficial.^8^, ^9^ Cancer immunotherapy drugs, including CTLA‐4, PD‐1 and PD‐L1 inhibitors, induce the reactivation of T cells for immune response to the tumor effect, thereby achieving an antitumor role.^10^ However, understanding tumor immune biology and inflammatory cell migration, as well as longitudinal changes of other complete blood count (CBC) parameters such as absolute lymphocyte count (ALC), platelet count (PLT) and absolute neutrophil count (ANC) play a crucial role in the development of newer generations of cancer immunotherapies and treatment strategies.^8^

It is widely accepted that, in addition to high PD‐L1 expression, tumor mutational burden and mismatch‐repair deficiency, as well as lymphocytes and other inflammatory cells also have a predictive role in immunotherapy response.^11^ A recent study clearly demonstrates that lymphopenia is indicative of poor prognosis in non‐small cell lung cancer (NSCLC) patients receiving immunotherapy.^12^, ^13^ Nonetheless, to date, immunotherapy is usually administered in advanced stage or metastatic lung carcinoma when the lymphocyte count is already expected to be low.^14^ Furthermore, based on a recent study, platelets are also greatly involved in immune regulation, tumor cell cross‐talk and tumor growth.^9^, ^10^ Accordingly, high PLT correlates with metastasis and reduced overall survival (OS).^9^ Moreover, platelets might play a crucial role in the inhibition of immune cell function as well, especially CD8 T cell function, and could be of importance in designing immunotherapy approaches with platelet inhibitors or engineered platelets.^9^, ^15^, ^16^, ^17^ Meanwhile, with regard to ANC, Parikh *et al*. found that metastatic NSCLC patients with high ANC were less likely to respond to immunotherapy than those with lower ANC.^18^

Collectively, CBC parameters serve as surrogate markers of inflammation and might reflect inflammation in the tumor microenvironment.^19^ In addition to immunotherapy, CBC parameters also influence chemotherapy and bisphosphonate tolerability in brain and bone metastatic patients, respectively.^20^ Accordingly, exploring the longitudinal changes in CBC parameters during cancer progression might provide useful information on expected drug efficacy, thereby assisting clinicians in the development of new treatment strategies. However, to date, to our knowledge, no data regarding changes in blood parameters during lung cancer progression and metastatic spread in a large and homogenous patient cohort has been reported, therefore, our cross‐sectional study aimed to explore the longitudinal changes in the aforementioned parameters in a cohort of brain and bone metastatic Caucasian patients.

## Methods

### Ethics statement

The present study was directed in accordance with the guidelines of the Helsinki Declaration of the World Medical Association. The national level ethics committee (Hungarian Scientific and Research Ethics Committee of the Medical Research Council, ETT‐TUKEB 23636‐2/2018, 23636/10/2018/EÜIG) approved the study. The need for individual informed consent for this retrospective study was waived. After clinical information was collected, patient identifiers were removed, and subsequently, patients could not be identified either directly or indirectly.

### Study population

In this single‐center, retrospective study, 986 consecutive lung cancer patients receiving standard of care therapy were evaluated between 2000 and 2016 at the National Koranyi Institute of Pulmonology, Budapest, Hungary. Of these, 818 patients were initially diagnosed either with brain or bone metastases while the remaining 168 patients diagnosed only with other distant organ metastases or no distant metastases until death served as a control group. Based on our inclusion and exclusion criteria cytologically or histologically verified lung adenocarcinoma (ADC), squamous cell carcinoma (SCC), small cell lung cancer (SCLC), and patients with other primary lung cancer (including large cell neuroendocrine carcinoma and large‐cell lung carcinoma) were included with available clinicopathological data and CBC reports. Of note, due to the limited number of SCLC patients included, no control group for this histopathological type was used. Patients with comorbidities, such as acute infection or sepsis which could have influenced the laboratory results were excluded from this analysis. Similarly, patients with high dose steroid treatment or with laboratory results less than three weeks after the end of chemotherapy were also excluded. Clinicopathological data included gender, age at time of primary tumor diagnosis, smoking history, tumor localization and histology, comorbidities and OS. In patients who underwent lung resection surgery, pathological TNM stage according to the Union for International Cancer Control (seventh edition) was recorded.^21^ Bone and brain metastases were diagnosed by CT scan, PET‐CT, and magnetic resonance imaging. OS was calculated from the diagnosis of brain or bone metastasis until death or last available follow‐up. Clinical follow‐up was closed on the 10^th^ of January 2019.

### Treatment

Based on the ESMO Clinical Practice Guidelines on Lung Cancer, patients were treated either by lung resection surgery including lobectomy, segmentectomy or pneumectomy, or by standard of care chemotherapy.^22^, ^23^ Drug administration was also performed in accordance with the contemporary guidelines and Hungarian health care financial regulations. Accordingly, ADC and SCC patients were treated either with a platinum‐based doublet regimen or in case of *EGFR* mutation or ALK translocation with EGFR‐TKI or ALK‐TKI, respectively. Of note, targeted therapeutic agents became part of the standard of care therapy at the beginning of 2015 in Hungary. Consequently, only 4.9% of all included patients could have received targeted therapy. The vast majority of patients with SCLC were treated either with a platinum‐etoposide doublet regimen, or a combination of cyclophosphamide, epirubicin, and vincristine (CEV). Patients with identified skeletal metastases received bisphosphonate therapy, while the management of patients presenting with brain metastases included whole‐brain radiotherapy, stereotactic radiosurgery or surgical metastasectomy. None of the patients received immunotherapy.

### Investigated CBC parameters

CBC was performed as part of the routine checkup using the same reference values during the whole follow‐up and the results were recorded according to: (i) the time of lung cancer diagnosis; (ii) diagnosis of brain or bone metastasis; and (iii) last available follow‐up usually less than one month before death. From the CBC we recorded the PLT, leukocyte count (WBC), ANC, and ALC. Ranges between 3.6–11.0 a for WBC, 1.8–7.5 giga per liter (G/L)G/L for ALC, and 140–400 G/L for PLT were considered normal values. Platelet‐to‐lymphocyte ratio (PLR) was calculated by dividing the absolute platelet numbers by the absolute lymphocyte numbers, while neutrophil‐to‐lymphocyte ratio (NLR) by dividing the neutrophil counts by the lymphocyte counts. In patients serving as the control group, only the CBC parameters at time of lung cancer diagnosis and the last available laboratory results were recorded. Longitudinal analyses were also performed by using both the mean values and the normal/abnormal ranges of the aforementioned CBC parameters. Abnormal low and high subgroups for each CBC parameter were defined based on the aforementioned reference values used by the medical laboratory where the blood tests were conducted. Accordingly, the lower and upper limits of the standard reference range were used as cutoff values when defining abnormal low and abnormal high subgroups.

### Statistical analysis

All statistical analyses were performed using the PASW Statistics 23.0 package (SPSS Inc., Chicago, IL, USA). Data distribution was verified by the Kolmogorov‐Smirnov normality test. Longitudinal analyses of CBC parameters were performed by the nonparametric Friedmann and Wilcoxon signed‐rank tests. The Chi‐square test was used to investigate associations between nominal variables. For univariate survival analysis, Kaplan–Meier curves and two‐sided log‐rank tests were used. Metric data are always shown as median or mean and corresponding range or, in case of OS, as median and corresponding 95% CI. Two‐sided *P*‐values less than 0.05 were considered statistically significant.

## Results

### Clinicopathological characteristics

A total of 986 lung cancer patients with brain and/or bone metastases, only other distant organ metastases or no distant metastases were included in this study: 435 females and 551 males (age range, 32–88 years; median, 60 years). Major clinicopathological data according to organ‐specific metastases are shown in Table 1. Based on the metastatic site, patients were divided into a control group and an experimental group. As a result of no significant differences in CBC parameters among the patients with no organ metastases and other distant organ metastasis at the time of lung cancer diagnosis and last follow‐up before death, these patients were grouped together serving as the control group (*n* = 168). In the experimental subcohort (*n* = 818), 352 (43%) and 466 (57%) cases were diagnosed with brain and bone metastases, respectively. Of note, *n* = 38 individuals from the patients’ subgroup with brain metastases also developed bone metastases in later stages and *n* = 85 individuals from the patients diagnosed with bone metastases developed brain metastases during progression. Concerning the genders, bone metastases were significantly more common among male patients while the incidence of brain metastases was higher among female patients (*P* = 0.006). Moreover, the occurrence of bone metastases was more frequent among elderly (age ≥ 65 years) patients while brain metastases appeared more frequently in the younger (age < 65 years) patients (*P* = 0.006).

**Table 1:** Clinicopathological features, overall survival (OS) and complete blood count (CBC) parameters according to organ‐specific metastases

(a)					
		Brain metastasis	Bone metastasis	Other type of distant metastasis	No distant metastasis
All patients		Count (n)			
Gender	Female	179	192	32	32
	Male	173	274	46	58
Age	<65	197	216	37	42
	≥65	155	250	41	48
Smoking	Non‐smoker	36	49	11	5
	Ex‐smoker	75	100	33	40
	Smoker	207	187	26	33
	Unknown	34	130	8	12
Histology	Adenocarcinoma	254	281	55	47
	Squamous cell carcinoma	47	110	18	38
	SCLC	50	70	5	5
	Other (LCNEC, LCC)	1	5	0	0
		Median OS (weeks)			
Survival	From date of primary tumor	63.8	66.4	168.0	165.0
	From date of metastasis	21.0	31.2	‐	‐

(b)				
		Diagnosis of primary tumor	Diagnosis of metastasis	Last follow‐up before death
CBC parameter		Mean (m)		
Brain metastasis	Platelet count (G/L)	317.04	298.77	307.53
	Leukocytes (G/L)	10.03	10.34	11.61
	Absolute neutrophil count	7.1	7.83	9.42
	Absolute lymphocyte count	1.94	1.56	1.19
	NLR	4.56	7.05	11.27
	PLR	197.3	259.75	335.43
Bone metastasis	Platelet count (G/L)	317.22	335.24	322.95
	Leukocytes (G/L)	9.35	9.97	12.43
	Absolute neutrophil count	6.44	7.33	10.01
	Absolute lymphocyte count	1.93	1.64	1.37
	NLR	3.78	6.1	11.54
	PLR	186.45	269.53	341.99
Other type of distant metastasis	Platelet count (G/L)	292.34	‐	311.66
	Leukocytes (G/L)	9.59	‐	11.77
	Absolute neutrophil count	6.57	‐	9.68
	Absolute lymphocyte count	1.99	‐	1.08
	NLR	4	‐	13.45
	PLR	172.47	‐	404.64
No distant metastasis	Platelet count (G/L)	299.95	‐	327.72
	Leukocytes (G/L)	8.97	‐	14.01
	Absolute neutrophil count	6.13	‐	11.59
	Absolute lymphocyte count	1.88	‐	1.29
	NLR	3.74	‐	14.33
	PLR	184.63	‐	415.36

CBC, complete blood count; LCC, large cell carcinoma; LCNEC, large cell neuroendocrine carcinoma; NLR, neutrophil‐to‐lymphocyte ratio; OS, overall survival; PLR, platelet‐to‐lymphocyte ratio; SCLC, small cell lung cancer.

### Longitudinal analysis

**Figure 1:**
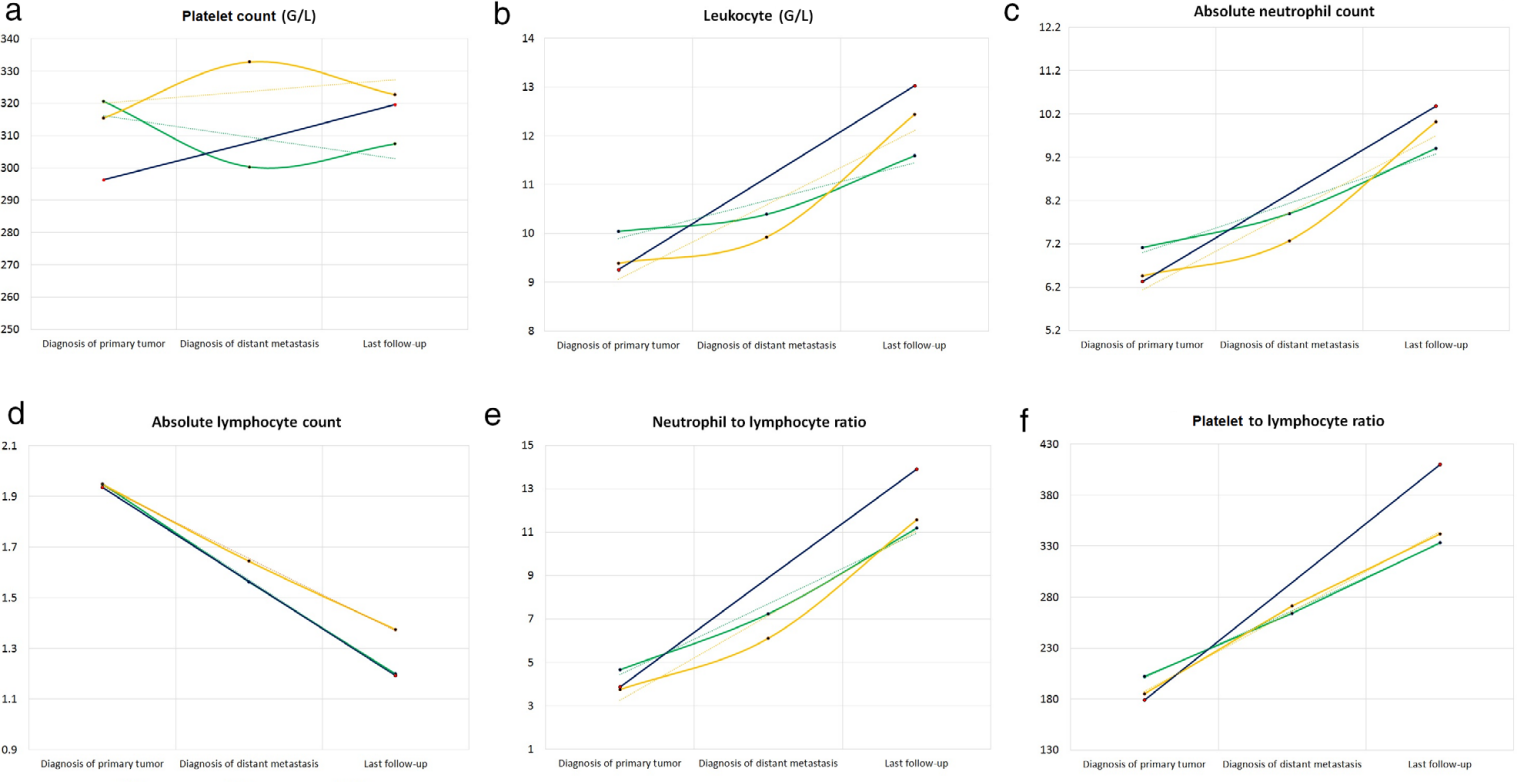
Longitudinal analysis of complete blood count (CBC) parameters and NLR and PLR ratios according to the date of diagnosis of the primary tumor, distant organ metastasis and last follow‐up. Plots illustrate how the occurrence of bone and brain metastases were related to the longitudinal courses of (**a**) platelet count (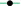) Brain, (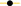) Bone, (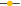) Control; (**b**) leukocyte count (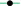) Brain, (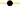) Bone, (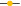) Control; (**c**) absolute neutrophil count (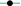) Brain, (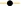) Bone, (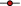) Control; (**d**) absolute lymphocyte counts (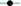) Brain, (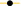) Bone, (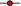) Control; (**e**) neutrophil to lymphocyte ratio (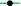) Brain, (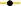) Bone, (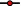) Control; and (**f**) platelet to lymphocyte ratio (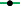) Brain, (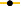) Bone, (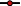) Control. The y‐axis corresponds to the value of the given CBC parameter, while the x‐axis shows the time frame according to the date of diagnosis of the primary tumor, distant organ metastasis and last follow‐up. The control group consisted of stage M0 patients and patients with other metastases. The dotted lines represent the linear trendline for each CBC parameter. *P*‐values for the distant organ metastases and assigned CBC parameters were obtained by the nonparametric Friedman test and Wilcoxon signed‐rank test.

We performed longitudinal analysis of the major CBC parameters according to the values recorded at the time of lung cancer diagnosis, diagnosis of metastases, and last follow‐up before death. Longitudinal courses of the CBC parameters, NLR and PLR according to the control and experimental groups irrespective of histopathological type of the tumor are shown in Fig 1. CBC parameters, such as WBC (Fig 1b) and ANC (Fig 1c) continuously and significantly increased during the longitudinal analysis, irrespective of metastatic sites, while the ALC (Fig 1d) showed continuous and significant descending mean values (*P* < 0.001). As for the mean PLT values (Fig 1a), significant changes were found during tumor progression in both patient subcohorts in the experimental group (brain metastasis, *P* < 0.001; bone metastasis, *P* = 0.043) and nonsignificant changes in the control group (*P* = 0.084). Regarding PLT levels, in case of patients with brain metastases, a descending‐ascending trendline (320.71 G/L vs. 300.21 G/L vs. 307.47 G/L), while in bone metastatic cases an ascending‐descending trendline (315.47 G/L vs. 332.92 G/L vs. 322.75 G/L) was observed. According to NLR and PLR, both ratios were significantly increased during the follow‐up (Fig 1e and f).

**Figure 2:**
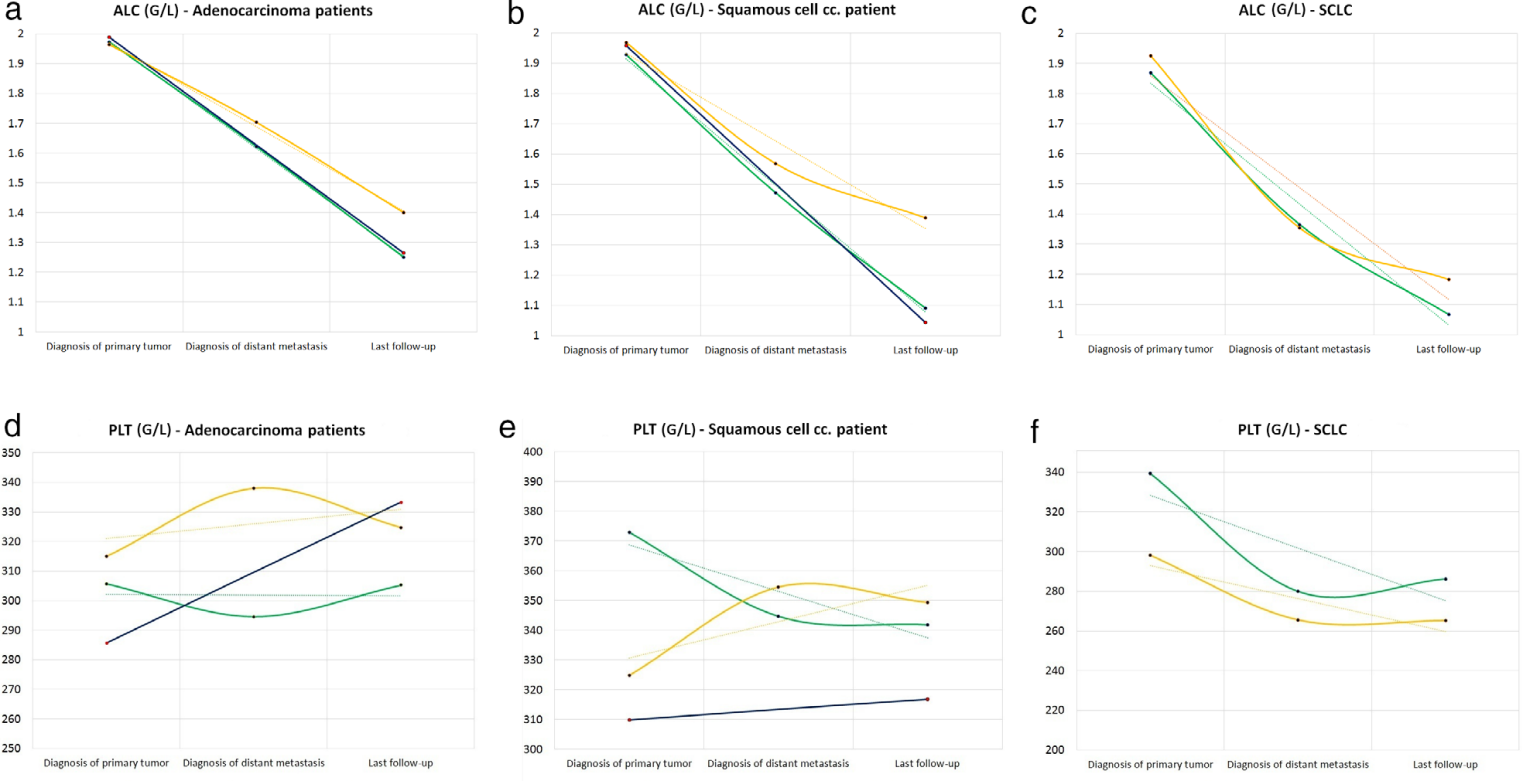
Longitudinal analysis of average absolute lymphocyte count (ALC) and platelet count (PLT) according to histopathological type of the primary tumor. Figures (**a**), (**b**), and (**c**) illustrate the longitudinal changes in average ALC in adenocarcinoma (ACC) patients, squamous cell carcinoma (SCC) patients and small cell lung cancer patients (SCLC), respectively (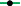) Brain, (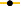) Bone, (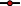) Control. Figures (**d**), (**e**), and (**f**) illustrate the longitudinal changes in average PLT in ADC patients, SCC patients and SCLC, respectively (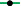) Brain, (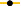) Bone, (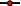) Control. The y‐axis corresponds to the value of the given complete blood count (CBC) parameter, while the x‐axis shows the time frame according to the date of diagnosis of the primary tumor, distant organ metastasis and last follow‐up. The control group consists of stage M0 patients and patients with other distant metastases. The dotted lines represent the linear trendline for each CBC parameter. *P*‐values for the distant organ metastases and assigned CBC parameters were obtained by the nonparametric Friedman test and Wilcoxon signed‐rank test.

Interestingly, when analyzing the ADC, SCC and SCLC patients separately, we found similar results in ALC (Fig 2a–c), but divergent trends for PLT according to the histopathological type (Fig 2d–f). ALC showed significantly descending values in all cases, irrespective of the type of metastasis and histopathological type. However, by performing longitudinal analysis of the mean PLT values at each time point, we found significantly descending‐ascending PLT levels in ADC patients with brain metastases (*P* = 0.041) and nonsignificantly ascending‐descending PLT levels in patients with bone metastases (*P* = 0.324). In contrast, in SCC, nonsignificantly descending‐slightly ascending values were found in cases of brain metastases, and significantly ascending‐descending PLT levels were found in bone metastatic patients (*P* = 0.001). Interestingly, in SCLC, both brain and bone metastatic patients had descending‐slightly ascending PLT levels (*P* = 0.006 and *P* = 0.177, respectively), and no ascending trend was observed after the diagnosis of the primary tumor.

**Figure 3:**
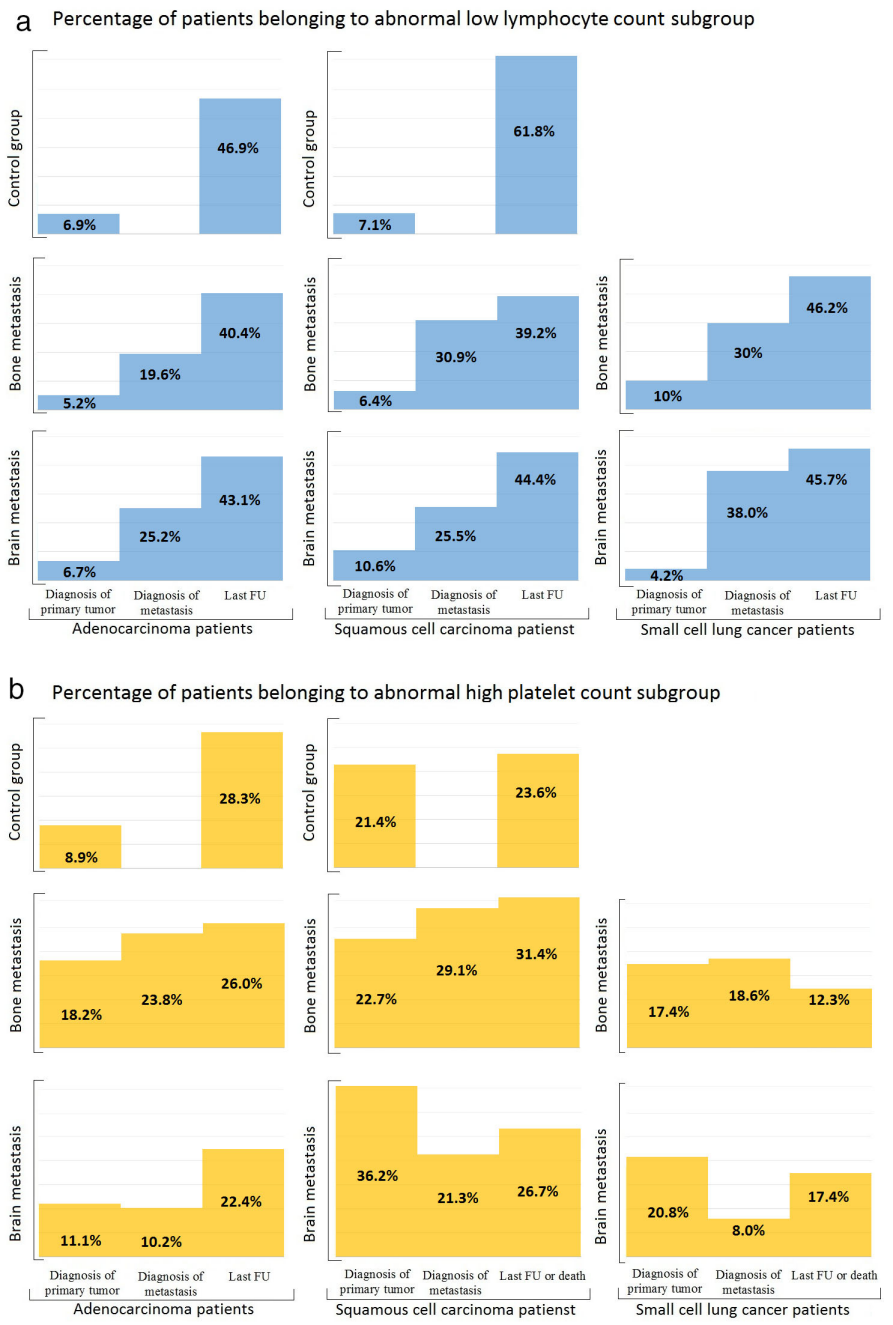
Longitudinal analysis of abnormal low and abnormal high subgroups of absolute lymphocyte count (ALC) and platelet count (PLT). (**a**) Percentage of patients belonging to the abnormal low ALC subgroup at the time of diagnosis of primary tumor, diagnosis of metastasis and last follow‐up before death according to histopathological type of the primary tumor and type of metastasis (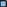) Low ALC. (**b**) Percentage of patients belonging to the abnormal high absolute PLT subgroup at the time of diagnosis of primary tumor, diagnosis of metastasis and last follow‐up before death according to histopathological type of the primary tumor and type of metastasis (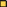) High PLT.

For the final step of the longitudinal analysis, in order to study the clinical relevance of the CBC parameters, patients were grouped based on the reference values of ALC and PLT (Fig 3 and Table S1). Accordingly, the percentage of patients belonging to the low abnormal ALC subgroup was increasing in all patient subgroups irrespective of the histopathological type of the tumor or type of metastasis, while the percentage of patients belonging to the high abnormal PLT subgroup was constantly increasing in ADC patients and decreasing in SCLC patients.

### Distinct effect of brain and bone metastases on CBC parameters, NLR and PLR values

**Figure 4:**
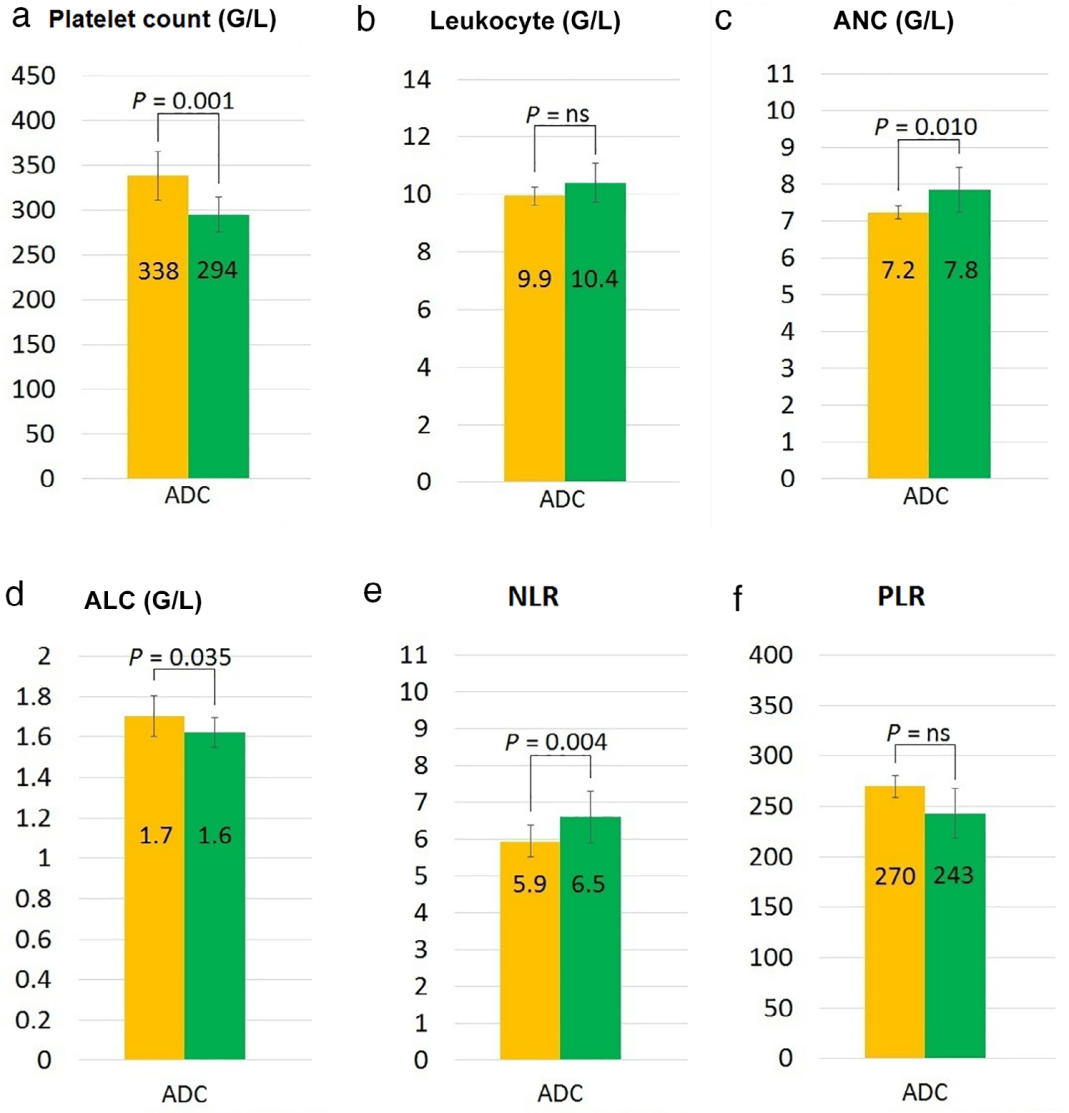
Average level of each complete blood count (CBC) parameter and NLR, PLR values at the diagnosis of the metastatic lesion according to distant organ metastases in adenocarcinoma (ADC) patients. The bar charts illustrate the differences in the average levels of CBC parameters between brain and bone metastatic lung ADC patients. Metastasis‐related subgroups were compared by using the nonparametric Mann–Whitney U test. (**a**) Platelet count (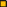) Bone metastasis, (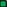) Brain metastasis; (**b)** Leukocyte (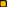) Bone metastasis, (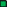) Brain metastasis; (**c**) Absolute neutrophil count (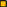) Bone metastasis, (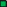) Brain metastasis; (**d**) Absolute lymphocyte count (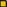) Bone metastasis, (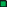) Brain metastasis; (**e**) Neutrophil to lymphocyte ratio (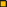) Bone metastasis, (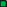) Brain metastasis; (**f**) Platelet to lymphocyte ratio (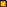) Bone metastasis, (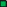) Brain metastasis.

As shown in Fig 4, we found significantly divergent average levels for PLT (*P* = 0.001), ANC (*P* = 0.010), ALC (*P* = 0.035), and NLR (*P* = 0.004) levels in brain metastatic versus bone metastatic ADC patients at the time of metastasis diagnosis. Furthermore, a visible, but not significant difference was also found in NLR (6.2 vs. 9 in bone vs. brain metastasis, respectively) and PLR (282 vs. 327 in bone vs. brain metastasis, respectively) values in SCC patients. According to other parameters, no significant differences were found regarding the histopathological type.

### Distinct effects of brain and bone metastases on overall survival

Fig S1 shows the OS of brain and bone metastatic patients according to ALC and PLT. With regard to the ALC, as expected, patients belonging to the high ALC subgroup had a nonsignificant, but visibly longer median OS than those having lower ALC at the diagnosis of brain metastasis, and a significantly longer OS in bone metastases. Accordingly, patients belonging to the high ALC subgroup (ALC ≥ 1.37 G/L) had a median OS of 28 weeks (vs. 17.7 weeks in the low ALC subgroup; *P* = 0.130; Fig S1a) in brain metastatic patients, and a median OS of 35 weeks (vs. 25.7 weeks in the low ALC subgroup; *P* = 0.0001; Fig S1c) in bone metastatic patients. Meanwhile, according to the PLT subgroups at the time of metastasis diagnosis, no significant difference was found in OS in case of brain metastatic patients (median OS, 18.7 vs. 24.2 weeks, respectively; *P* = 0.446; Fig S1b). On the contrary, in bone metastatic patients OS was significantly higher among patients with low PLT (vs. high PLT; median OS, 36.5 vs. 26.1 weeks; *P* = 0.007; Fig S1d). OS of patients with brain, and bone metastases according to histopathological type of the primary tumor is shown in Fig S2.

## Discussion

The prognosis for patients with extensive‐stage lung cancer with known brain or bone metastases is still poor with a median survival of less than one year; however, with the advances in molecular targeted therapy and immunotherapy, therapeutic options have increased vastly over the past decade.^6^, ^7^, ^18^ In recent years, numerous CBC parameters, such as PLT, ALC and ANC, have been described as easily accessible biomarkers that might predict response to immunotherapy; however, to date, no comprehensive study has been performed on the longitudinal changes of these parameters during cancer progression and metastatic spread.^8^, ^9^, ^10^, ^15^, ^16^, ^17^, ^18^, ^24^, ^25^, ^26^

First, we analyzed the association of major clinicopathological characteristics and occurrence of brain or bone metastases. Accordingly, we found that the incidence of brain metastases was higher among female patients, whereas bone metastases appeared more frequently among male patients. Although, to date, no significant association has been reported by others between organ preference and gender, it is suspected that organ preference is most likely attributable to a combination of hormonal regulation and underlying biology related to sexual dimorphism.^27^, ^28^, ^29^, ^30^ However, our results regarding age distribution and metastatic spread are in line with others since we also found that brain metastases appeared more frequently in the younger population and bone metastases among elderly patients.^30^, ^31^, ^32^ A possible explanation might be that brain metastases are associated with the angiogenic microenvironment, and the cerebrovascular microenvironment factors of young patients may be better than those of older patients, while in the case of bone metastases the more favorable bone marrow microenvironment for metastatic spread and the higher incidence of osteoporosis in elderly patients might contribute to the higher incidence of bone metastases.^31^, ^33^, ^34^

Next, we focused on the longitudinal changes of the measured CBC parameters during cancer progression and metastatic spread, and found significant changes with regard to all parameters in the experimental group. According to the longitudinal analysis, we found significantly decreasing ALC in all patient subgroups irrespective of histopathological and metastatic types. Lymphocytes play a prominent role in tumor‐related immunology and lymphopenia has been reported to be associated with poor prognosis in many solid tumors including NSCLC, melanoma, gastric‐, breast‐, and colon cancers.^12^, ^35^, ^36^ Cho *et al*. investigated the effects of lymphopenia on OS and progression‐free survival (PFS) in immunotherapy treated patients and found that lymphopenia within 0–2 months from the initiation of immunotherapy also showed significantly inferior OS and PFS.^12^ Accordingly, changes in ALC should be carefully evaluated in patients who are future candidates for immunotherapy. With regard to other CBC parameters, divergent trends were observed for PLT in cases of brain and bone metastases according to both the histopathological type and localization of metastases. In NSCLC cases, descending‐ascending and ascending‐descending trendlines were found in brain and bone metastatic patients, respectively. However, the average PLT levels were significantly higher in bone metastatic ADC patients, and nonsignificantly higher in bone metastatic SCC patients than those with brain metastases. Our results are in line with an Asian study, performed on a cohort of Chinese patients, which also identified the phenomenon of pulmonary adenocarcinoma‐associated thrombocytosis in bone metastatic patients.^37^ With regard to bone metastases, the role of platelets in skeletal metastases and in bone remodeling including osteoclastogenesis and bone resorption remains controversial. However, although the mechanisms are not yet fully understood, blocking platelet aggregation has been reported to inhibit the progression of skeletal metastases.^38^ A possible explanation might be that since the appearance of bone metastases is influenced by the bone remodeling process, cytokines and growth factors released upon platelet activation might play a key role in skeletal tumor growth.^38^ Interestingly, in our SCLC patients, the average PLT was higher among patients with brain metastases, although the difference was not significant. This might be due to the relatively small sample size of SCLC patients, or to the fact that thrombocytosis may only correlate with one type of bone metastasis (osteoblastic, osteolytic or mixed) and we had no information on the type of skeletal metastasis.^37^ When analyzing the patient distribution belonging to each PLT subgroup based on the normal range of PLT, we also found that the percentage of patients belonging to the abnormal high subgroup was constantly increasing during the follow‐up with respect to ADC patients and decreasing in SCLC patients. An elevated platelet count has been found to be associated with progression in other types of cancer, including breast cancer and pancreatic cancer, while the nonelevated platelet count in SCLC might be due to the relatively rapid progression and short OS in this type of lung cancer.^39^, ^40^ The average levels of WBC and ANC were constantly increasing during the whole follow‐up irrespective of histopathological type and both had higher values at the diagnosis of metastasis in brain metastatic NSCLC patients. Our findings are supported by a preclinical mouse model study which also found altered WBC, ANC and PLT levels in breast cancer metastatic mice.^41^

Finally, we investigated the prognostic role of ALC and PLT levels at the time of diagnosis of metastasis. Brain metastatic patients with low ALC and high PLT had significantly shorter OS than those having higher ALC and lower PLT values. Interestingly, no significant differences were found in OS related neither to ALC and PLT levels in bone metastatic patients, although the OS was visibly higher in case of high ALC. Our findings are in accordance with other studies which report that both ALC and PLT have a prognostic role in various types of cancer.^35^, ^40^, ^42^ Regarding the type of metastasis, we found significantly higher OS in ADC patients with bone metastasis than those with brain metastasis.

Our findings are of high clinical importance, since, to date, this is the first study on the longitudinal changes of CBC parameters in brain and bone metastatic lung cancer patients. Immunotherapy has revolutionized cancer therapy and checkpoint inhibitors have demonstrated unprecedented rates of durable responses in some of the most difficult to treat cancers, but there still is an unmet need of identifying patients who will best respond to therapy.^43^ Moreover, immunotherapy is usually administered in advanced stage or metastatic lung carcinoma as a second‐ or third‐line treatment option, when the response rates of patients are lower and, more often than not, only a small subset of patients respond favorably and durably.^14^ The continuously descending ALC and ascending ANC might contribute to low response rates. The mechanism of anti‐PD1 antibodies is dependent on the activity of functional T lymphocytes, thus the efficacy of anti‐PD1 antibodies are compromised in patients with lymphopenia.^44^ Meanwhile, high ANC is also associated with lower response rates to newly administered immunotherapeutic agents, most likely due to an already intense baseline tumor‐associated inflammatory reaction.^18^ As for other CBC parameters, platelets release factors that support tumor growth, metastatic spread and also form heterotypic aggregates with tumor cells, which can provide an immune‐evasive advantage. Hence, one of their critical roles may be the inhibition of immune cell function that can negatively impact the response rates to immunotherapy.^9^ Accordingly, the earlier discussed high PLT levels in advanced stage brain and bone metastatic lung cancer patients (especially in case of bone metastasis) predicts poor response rates for these patients. The divergent trends found in case of brain and bone metastases might be suggestive for more individualized treatment strategies for these patients. Collectively, ALC, ANC and PLT trendlines in brain and bone metastatic lung cancer patients are suggestive of a poor response to immunotherapy. However, analyzing the divergent trends found in our cohort of the aforementioned CBC values might help lay the framework for new therapeutic strategies with regard to time of administration.

Our study had certain limitations. First, it was retrospective with given limitations in interpreting its results. Another potential limitation was that no precise information was available on the exact type of bone metastases (osteoblastic, osteolytic or mixed) or in ADC cases mutational status of the primary tumor (such as *KRAS*, *EGFR* or *ALK*). Moreover, as well as the absence or presence of brain or bone metastases, CBC parameters might also have been influenced by a number of other factors, such as infection, rheumatic autoimmune disease and coronary heart disease. Although we used a relatively large and homogenous patient cohort, patients were included over a long period of time, and hence multiple treatment methods were administered which may have influenced the prognosis and CBC parameters. Furthermore, due to the fact that modern imaging methods, such as FDG PET‐CT, were not utilized as standard examination methods in the early 2000s, we were unable to diagnose asymptomatic or micrometastases, which may have led to an underestimation of the presence of metastases in some of the patients included in the study. Collectively, taking into account all the aforementioned potential study limitations, caution is needed when interpreting the results of our study.

In conclusion, this study highlights the longitudinal changes in CBC parameters during brain and bone metastatic spread in lung cancer patients. The ascending mean values for PLT and ANC, as well as the descending values for ALC, might be suggestive of a poor response to second‐ or third‐line immunotherapy in advanced stages of lung cancer. Altogether, the current study might help to improve patient selection and treatment strategies for brain and bone metastatic lung cancer patients in the future.

## Disclosure

No authors report any conflict of interest.

## Supporting information


**Supplementary Figure S1** Kaplan–Meier survival curves for overall survival (OS) from metastasis diagnosis in brain and bone metastatic lung cancer patients according to absolute lymphocyte count (ALC) and platelet count (PLT) at the time of diagnosis of metastasis.
(**a**) Overall survival (OS) of brain metastatic lung cancer patients according to the median absolute lymphocyte count (ALC) at the time of initial diagnosis of brain metastasis (median OS, ALC < 1.37 g/L vs. ALC ≥ 1.37 g/L, 17.4 vs. 28 weeks, *P* = 0.130, *n* = 264, log‐rank test). (**b**) OS of brain metastatic patients with smaller platelet count (PLT) (<290 g/L) was nonsignificantly shorter compared to those with high PLT (≥290 g/L) (median OS, 18.7 vs. 24.2 weeks, *P* = 0.446, *n* = 264, log‐rank test). (**c**) OS of bone metastatic patients with ALC lower than the median value (<1.49) (vs. higher ALC [≥1.49], median OS, 25.7 vs. 35 weeks, *P* = 0.0001, *n* = 459, log‐rank test). (**d**) OS of bone metastatic lung cancer patients with lower PLT (<307 g/L) (vs. higher PLT [≥307 g/L], median OS, 36.5 vs. 26.1 weeks, *P* = 0.007, *n* = 459, log‐rank test).

**Supplementary Figure S2** Kaplan–Meier survival curves for overall survival (OS) from metastasis diagnosis in brain and bone metastatic lung cancer patients according to the histopathological type of the primary tumor.
(**a**) Overall survival (OS) of lung adenocarcinoma patients with brain metastasis was significantly shorter compared to those with bone metastasis (median OS, 27.8 vs. 36.2 weeks, *P* = 0.033, *n* = 482, log‐rank test). (**b**) Visible, but not statistically significant differences in OS have been observed for squamous cell carcinoma patients with brain metastasis versus patients with bone metastasis (median OS were 10.2 vs. 26.1 weeks, respectively *P* = 0.05, *n* = 141, log‐rank test). (**c**) OS of small cell lung cancer patients with brain metastasis versus bone metastasis (median OS, 15 vs. 15.2 weeks, *P* = 0.884, *n* = 67, log‐rank test).

**Supplementary Table S1** Percentage of patients belonging to abnormal low, normal and abnormal high subgroups according to absolute lymphocyte count (ALC) and platelet count (PLT) percentage at different time points during disease progression.
