# Supplementary figures and images for: Longitudinal analysis of complete blood count parameters in advanced‐stage lung cancer patients
Source: Thorac Cancer. 2020 Sep 17;11(11):3193–204. doi: 10.1111/1759-7714.13642 (PMC7605999; doi:10.1111/1759-7714.13642)

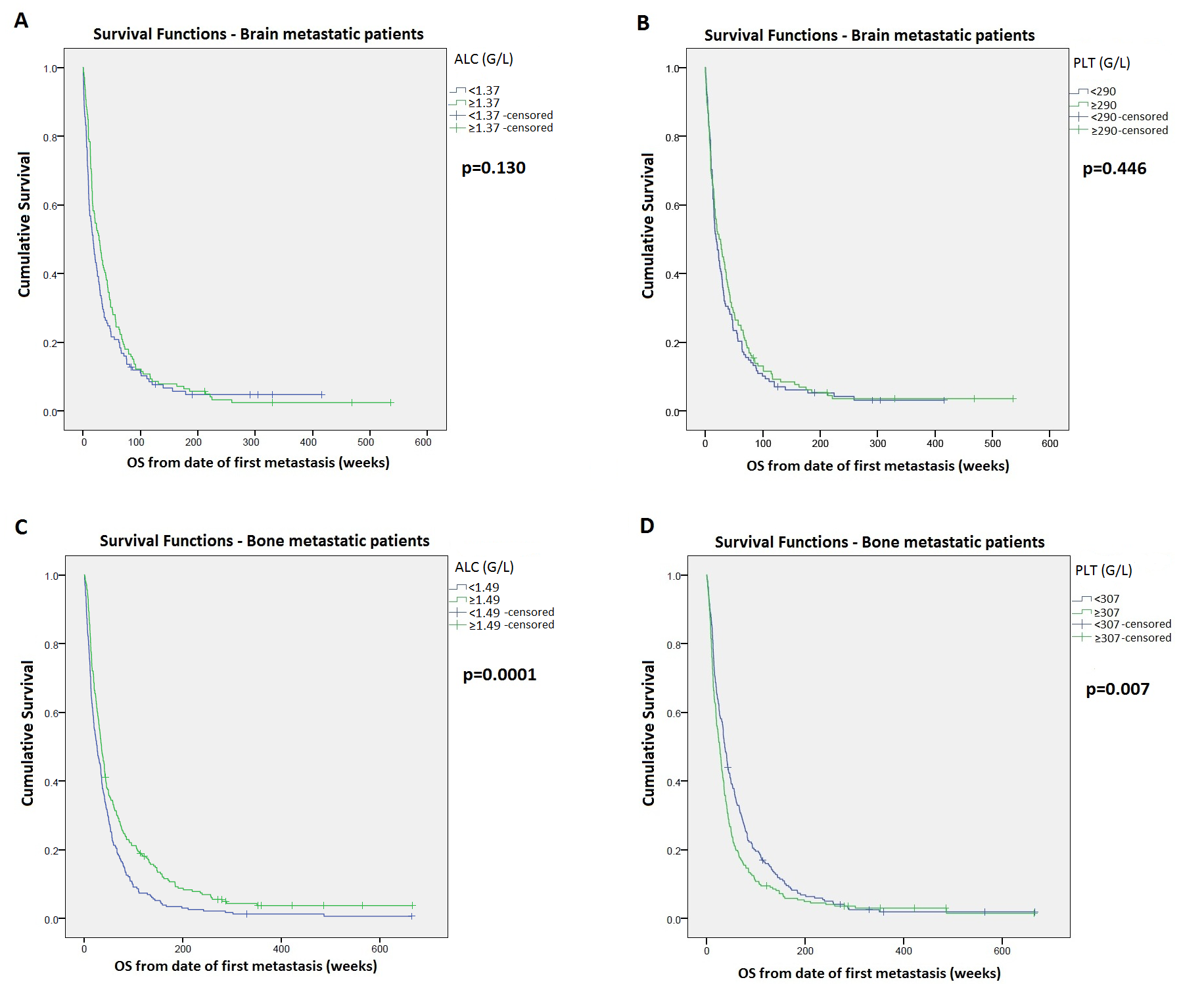

Supplement: Supplementary file 1 — Supplementary Figure S1 Kaplan–Meier survival curves for overall survival (OS) from metastasis diagnosis in brain and bone metastatic lung cancer patients according to absolute lymphocyte count (ALC) and platelet count (PLT) at the time of diagnosis of metastasis. (a) Overall survival (OS) of brain metastatic lung cancer patients according to the median absolute lymphocyte count (ALC) at the time of initial diagnosis of brain metastasis (median OS, ALC < 1.37 g/L vs. ALC ≥ 1.37 g/L, 17.4 vs. 28 weeks, P = 0.130, n = 264, log‐rank test). (b) OS of brain metastatic patients with smaller platelet count (PLT) (<290 g/L) was nonsignificantly shorter compared to those with high PLT (≥290 g/L) (median OS, 18.7 vs. 24.2 weeks, P = 0.446, n = 264, log‐rank test). (c) OS of bone metastatic patients with ALC lower than the median value (<1.49) (vs. higher ALC [≥1.49], median OS, 25.7 vs. 35 weeks, P = 0.0001, n = 459, log‐rank test). (d) OS of bone metastatic lung cancer patients with lower PLT (<307 g/L) (vs. higher PLT [≥307 g/L], median OS, 36.5 vs. 26.1 weeks, P = 0.007, n = 459, log‐rank test). [file TCA-11-3193-s001.png]

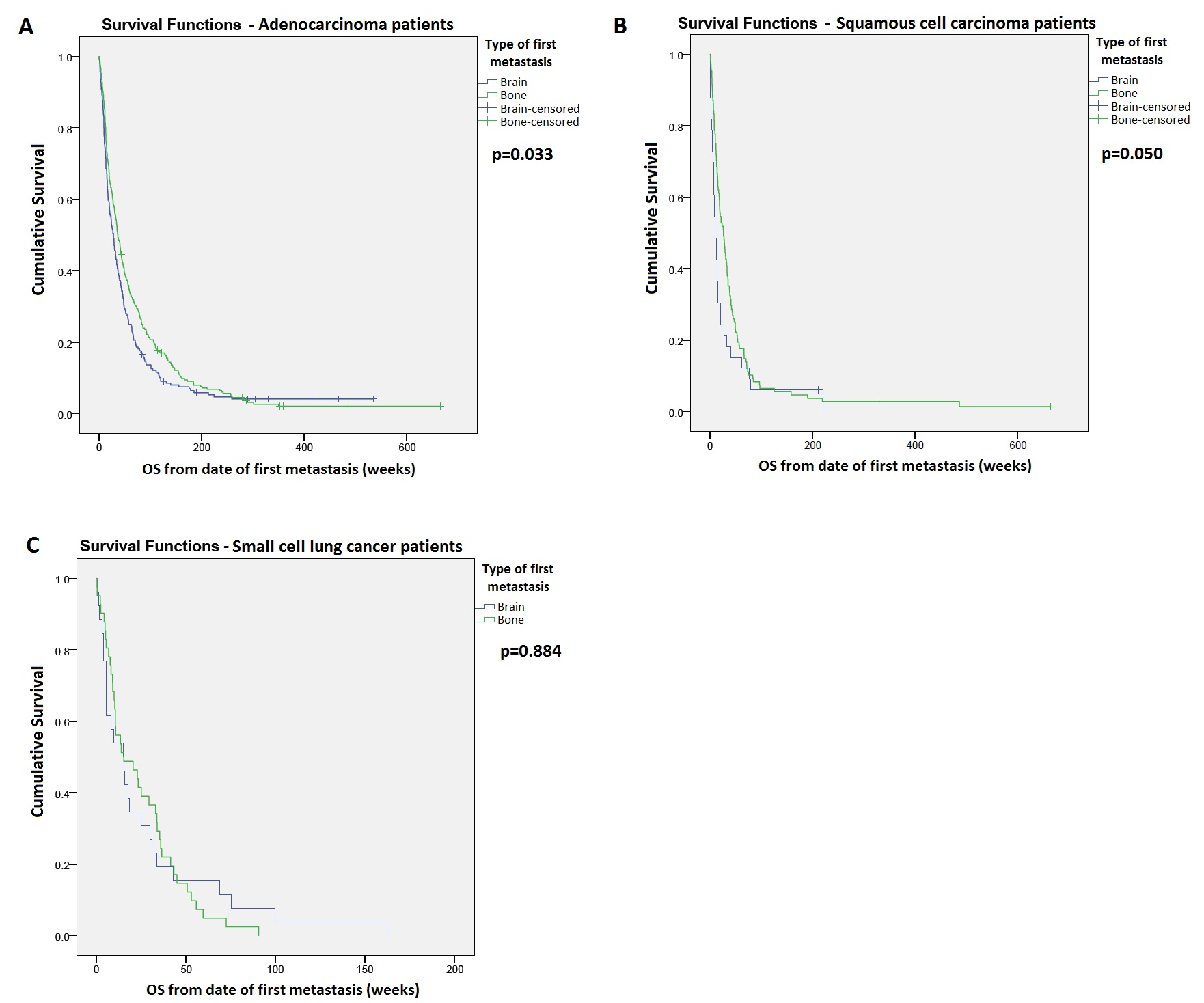

Supplement: Supplementary file 2 — Supplementary Figure S2 Kaplan–Meier survival curves for overall survival (OS) from metastasis diagnosis in brain and bone metastatic lung cancer patients according to the histopathological type of the primary tumor. (a) Overall survival (OS) of lung adenocarcinoma patients with brain metastasis was significantly shorter compared to those with bone metastasis (median OS, 27.8 vs. 36.2 weeks, P = 0.033, n = 482, log‐rank test). (b) Visible, but not statistically significant differences in OS have been observed for squamous cell carcinoma patients with brain metastasis versus patients with bone metastasis (median OS were 10.2 vs. 26.1 weeks, respectively P = 0.05, n = 141, log‐rank test). (c) OS of small cell lung cancer patients with brain metastasis versus bone metastasis (median OS, 15 vs. 15.2 weeks, P = 0.884, n = 67, log‐rank test). [file TCA-11-3193-s002.jpg]
