# Supplementary material for: Longitudinal analysis of complete blood count parameters in advanced‐stage lung cancer patients
Source: Thorac Cancer. 2020 Sep 17;11(11):3193–204. doi: 10.1111/1759-7714.13642 (PMC7605999; doi:10.1111/1759-7714.13642)
Supplement: Supplementary file 3 — Supplementary Table S1 Percentage of patients belonging to abnormal low, normal and abnormal high subgroups according to absolute lymphocyte count (ALC) and platelet count (PLT) percentage at different time points during disease progression. [file TCA-11-3193-s003.docx]

**Supplementary Table 1**. Percentage of patients belonging to abnormal low, normal and abnormal high subgroups according to absolute lymphocyte count (ALC) and platelet count (PLT) percentage at different time points during disease progression.

|  | | Absolute lymphocyte count^a^ | | | | Platelet count^b^ | | | |
| --- | --- | --- | --- | --- | --- | --- | --- | --- | --- |
| Patients | | PLT reference groups | Diagnosis of primary tumor | Diagnosis of metastasis | Last FU | ALC reference group | Diagnosis of primary tumor | Diagnosis of metastasis | Last FU |
| ADC patients | Brain metastasis | Low ALC^c^ | 6.7% | 25.2% | 43.1% | Low PLT^f^ | 1.2% | 2.8% | 8.1% |
|  |  | Normal ALC^d^ | 90.1% | 71.7% | 55.5% | Normal PLT^g^ | 87.7% | 87% | 69.5% |
|  |  | High ALC^e^ | 3.2% | 3.1% | 1.4% | High PLT^h^ | 11.1% | 10.2% | 22.4% |
|  | Bone metastasis | Low ALC^c^ | 5.2% | 19.6% | 40.4% | Low PLT^f^ | 0.7% | 2.8% | 7.5% |
|  |  | Normal ALC^d^ | 93.3% | 78.6% | 58.4% | Normal PLT^g^ | 81.1% | 73.3% | 66.5% |
|  |  | High ALC^e^ | 1.5% | 1.8% | 1.2% | High PLT^h^ | 18.2% | 23.8% | 26% |
|  | Control group^i^ | Low ALC^c^ | 6.9% | - | 46.9% | Low PLT^f^ | 3% | - | 10.1% |
|  |  | Normal ALC^d^ | 91.1% | - | 52.1% | Normal PLT^g^ | 88.1% | - | 61.6% |
|  |  | High ALC^e^ | 2% | - | 1% | High PLT^h^ | 8.9% | - | 28.3% |
| SCC patients | Brain metastasis | Low ALC^c^ | 10.6% | 25.5% | 44.4% | Low PLT^f^ | 4.3% | 6.4% | 6.7% |
|  |  | Normal ALC^d^ | 89.4% | 74.5% | 55.6% | Normal PLT^g^ | 59.6% | 72.3% | 66.7% |
|  |  | High ALC^e^ | 0% | 0% | 0% | High PLT^h^ | 36.2% | 21.3% | 26.7% |
|  | Bone metastasis | Low ALC^c^ | 6.4% | 30.9% | 39.2% | Low PLT^f^ | 0.9% | 0.9% | 2.9% |
|  |  | Normal ALC^d^ | 91.8% | 66.4% | 57.9% | Normal PLT^g^ | 76.4% | 70% | 65.7% |
|  |  | High ALC^e^ | 1.8 | 2.7% | 2.9% | High PLT^h^ | 22.7% | 29.1% | 31.4% |
|  | Control group^i^ | Low ALC^c^ | 7.1% | - | 61.8% | Low PLT^f^ | 5.4% | - | 5.5% |
|  |  | Normal ALC^d^ | 91.1% | - | 36.4% | Normal PLT^g^ | 73.2% | - | 70.9% |
|  |  | High ALC^e^ | 1.8% | - | 1.8% | High PLT^h^ | 21.4% | - | 23.6% |
| SCLC patients | Brain metastasis | Low ALC^c^ | 4.2% | 38% | 45.7% | Low PLT^f^ | 2.1 | 0% | 10.9% |
|  |  | Normal ALC^d^ | 95.8% | 60% | 54.3% | Normal PLT^g^ | 77.1% | 92% | 71.7% |
|  |  | High ALC^e^ | 0% | 2% | 0% | High PLT^h^ | 20.8% | 8% | 17.4% |
|  | Bone metastasis | Low ALC^c^ | 10% | 30% | 46% | Low PLT^f^ | 4.3% | 8.6% | 13.8% |
|  |  | Normal ALC^d^ | 88.6% | 70% | 53% | Normal PLT^g^ | 78.3% | 72.9% | 73.8% |
|  |  | High ALC^e^ | 1.4% | 0% | 0% | High PLT^h^ | 17.4% | 18.6% | 12.3% |

^a^Percentage of patients belonging to each group according to PLT and associated reference values, ^b^Percentage of patients belonging to each group according to ALC and associated reference values, ^c^ALC% < 1.0 G/L, ^d^ALC% 1.0-4.0 G/L ^e^ALC% > 4.0 G/L, ^f^PLT < 142 G/L, ^g^PLT 142-424 G/L, ^h^PLT > 424 G/L, ^i^The control group consists of patients with metastases other than bone or brain and patients without distant organ metastases.

ALC: Absolute lymphocyte count

PLT: Platelet count

ADC: Adenocarcinoma

SCC: Squamous cell carcinoma

SCLC: Small cell lung cancer

FU: Follow-Up
